# Ectopic Expression of *FvVND4c* Promotes Secondary Cell Wall Thickening and Flavonoid Accumulation in *Fragaria vesca*

**DOI:** 10.3390/ijms24098110

**Published:** 2023-04-30

**Authors:** Bei Zhang, Xiaofei Dang, Hao Chen, Tian Li, Fangjie Zhu, Shingo Nagawa

**Affiliations:** 1 College of Horticulture, Fujian Agriculture and Forestry University (FAFU), Fuzhou 350002, China; 2 College of Life Science, Fujian Agriculture and Forestry University (FAFU), Fuzhou 350002, China; 3 College of Future Technology, Fujian Agriculture and Forestry University (FAFU), Fuzhou 350002, China; 4 Fujian Agriculture and Forestry University–University of California, Riverside, Joint Center for Horticultural Biology and Metabolomics, Haixia Institute of Science and Technology, Fujian Agriculture and Forestry University, Fuzhou 350002, China

**Keywords:** secondary cell wall, flavonoid, *FvVND4c*, *FvMYB46*, *Fragaria vesca*

## Abstract

Secondary cell wall (SCW) thickening has a significant effect on the growth and development of plants, as well as in the resistance to various biotic and abiotic stresses. Lignin accounts for the strength of SCW. It is synthesized through the phenylpropanoid pathway that also leads to flavonoid synthesis. The coupling strategies for lignin and flavonoid syntheses are diverse in plants. How their syntheses are balanced by transcriptional regulation in fleshy fruits is still unclear. The diploid strawberry (*Fragaria vesca*) is a model for fleshy fruits research due to its small genome and wide scope of genetic transformation. SCW thickening is regulated by a multilevel transcriptional regulatory network wherein vascular-related NAC domains (VNDs) act as key regulators. In this study, we systematically characterized VNDs in *Fragaria vesca* and explored their functions. The overexpression of *FvVND4c* in diploid strawberry fruits resulted in SCW thickening and fruit color changes accompanied with the accumulation of lignin and flavonoids. Genes related to these phenotypes were also induced upon *FvVND4c* overexpression. Among the induced genes, we found *FvMYB46* to be a direct downstream regulator of *FvVND4c*. The overexpression of *FvMYB46* resulted in similar phenotypes as *FvVND4c*, except for the color change. Transcriptomic analyses suggest that both FvVND4c and FvMYB46 act on phenylpropanoid and flavonoid biosynthesis pathways, and induce lignin synthesis for SCW. These results suggest that FvVND4c and FvMYB46 cooperatively regulate SCW thickening and flavonoid accumulation in *Fragaria vesca*.

## 1. Introduction

Cell walls are unique cellular structures of plants, which protect plants from environmental stresses and promote directed growth [1,2]. Plant cell walls are classified into two types. The primary cell wall (PCWs), mainly composed of cellulose, hemicelluloses, and pectin, are dynamic structures that support the growth of plant cells and are fundamental for plant morphogenesis [3,4]. The secondary cell walls (SCW), mainly composed of cellulose, hemicellulose, and lignin, are deposited after the expansion of the PCW and ultimately provide support and rigidity to the plants [5].

The formation of the SCW is a complex process regulated by several gene co-expression networks, in which NAC and MYB are the master regulatory factors. In Arabidopsis, several NAC transcription factors (TFs) represent the first-layer switches that are able to turn on the entire SCW biosynthetic program. The NAC factors include the vascular-related NAC domains (VND1 to VND7) and the NAC secondary wall-thickening promoting factors (NST1, NST2, and NST3) [6,7,8]. All of these *VND* transcription factors are preferentially expressed in developing vascular tissues, although with different expression patterns. The ectopic overexpression of *VNDs* activates the genes for the biosynthesis of cellulose, xylan and lignin, thus causing the ectopic deposition of SCW [9,10,11]. MYB46 and MYB83 act as the secondary layer of master switches of SCW biosynthesis [6,7,8]. In Arabidopsis, *AtMYB46* and its closest functional ortholog *AtMYB83* are preferentially expressed in xylem tissues. Their overexpression induces the ectopic deposition of SCW [12,13]. Conversely, double T-DNA knockout mutations of *AtMYB83* and *AtMYB46* cause SCW loss in vessels and an arrest in plant growth [13]. Furthermore, AtVNDs and/or AtNSTs, the first layer of master regulators of the differentiation of SCW, directly bind to the promotors of *AtMYB46* and *AtMYB83* [12,13,14,15]. Thus, MYB46 and MYB83 act as the secondary layer of master switches of SCW biosynthesis.

Lignin is one of the main components of SCW, providing structural support, water transport capabilities and defense against microorganisms in vascular plants [16]. Lignin biosynthesis starts with the general phenylpropanoid pathway, which also generates precursors for flavonoids [17,18]. Flavonoids exhibit beneficial effects on plant growth and human health. Many studies have found that the biosyntheses of flavonoids and lignin are linked through transcription regulatory networks [19,20,21,22]. Recently, a study characterized the Ripening Inducing Factor (FaRIF), an NAC TF that regulates genes involved in cell wall degradation and the phenylpropanoid pathway. FaRIF controls strawberry fruit firmness and levels of anthocyanin and lignin. The ripe *FaRIF*-silenced fruits showed increased lignification and cell wall metabolism, but delayed ripening with increased fruit firmness and decreased red colorization in the fruits. In contrast, the overexpression of *FaRIF* induced cell wall degradation and flavonoid accumulation [23]. Thus, the regulation of cell wall formation and flavonoid accumulation in fleshy fruit seems to be interconnected, but the molecular mechanisms behind the interconnection are yet unclear.

Strawberry is one of the most popular fleshy fruits because of its unique flavor and fragrance [24,25]. Strawberry fruits easily soften and rot after ripening, resulting in a short shelf life and high storage and transportation costs, which greatly limit the development of strawberry industry. Therefore, the research on the formation of lignin and flavonoids biosynthesis in strawberry fruit provides a basis for the improvement of fruit quality. *Fragaria vesca*, a diploid woodland strawberry, is emerging as a model for the cultivated octoploid strawberry as well as for the *Rosaceae* family. *F. vesca* has multiple merits: a small and sequenced genome, diploidy (2n = 14, 240 Mb genome), small stature, a short life cycle, facile cultivation and a genetic transformation ability [26]. Among *F. vesca* accessions, the seventh inbred line “Yellow Wonder” (YW5AF7) develops white fruit, including white receptacles and white achenes, and is frequently utilized in research on color observations [27]. Comparing the genomic sequences of three *F. vesca* accessions—the red fruit “Ruegen” (‘Rügen’), and the white fruits “Hawaii 4” and “Yellow Wonder”—has identified a candidate SNP in *FvMYB10* that is responsible for the white color [28].

This study identified FvVND4c, an NAC family TF, as one of the master regulators of SCW formation and flavonoid accumulation in *F. vesca*. The transient overexpression of *FvVND4c* promoted ectopic SCW thickening and fruit color changes by inducing lignin biosynthesis and flavonoid accumulation. FvVND4c regulates genes associated with SCW thickening and flavonoid biosynthesis, including *FvMYB46*, the overexpression of which results in similar phenotypes. In addition, transcriptome analysis showed that FvVND4c and FvMYB46 both acted on phenylpropanoid and flavonoid biosynthesis. Furthermore, our results suggest that FvVND4c may directly bind to the promoters to regulate *FvMYB46*. Our findings demonstrate that FvVND4c positively regulates SCW thickening and flavonoid accumulation through FvMYB46. The insights also provide further understanding of the transcriptional regulatory mechanisms underlying the coordination of the biosynthesis of flavonoids and lignin.

## 2. Results

### 2.1. Expression Patterns of SCW-Related NACs in Fragaria vesca

**Figure 1:**
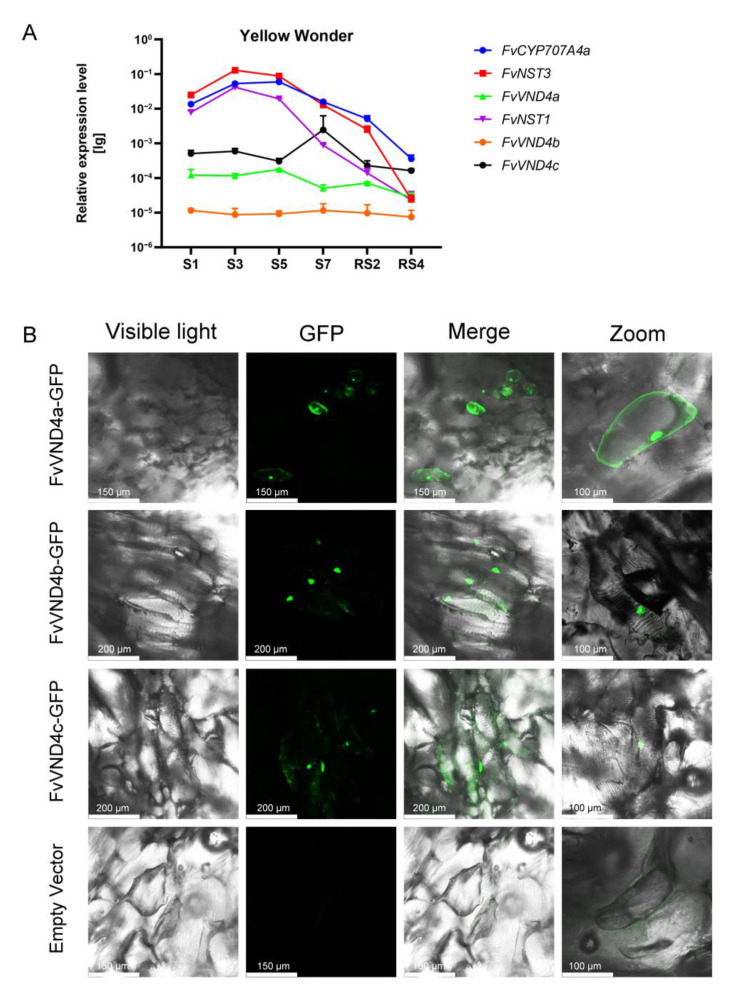
Expression patterns and subcellular localization of *FvVND4s*. (**A**) Expression levels of *FvVND4s* during strawberry fruit development; *FvCYP7074a* is a reference gene for early stages of fruit development [32]. (**B**) Subcellular localization of *FvVND4s* was determined by transient expression of the FvVND4s-GFP fusion protein in strawberry fruit cells. The images of bright field channel and GFP channel, and merged images of bright field and GFP channels, are shown.

Our previous study identified six *F. vesca* VND/NST candidate genes from the Strawberry Genomic Resources database (http://bioinformatics.towson.edu/strawberry/, accessed on 1 November 2018. FvNST1b in the NST cluster is closest to AtNST1, which has been proven to regulate SCW thickening [29]. FvNST3 has a similar function to FvNST1b. However, FvNST1a is likely a pseudogene because its expression is not detected in any plant developmental stages [29]. In order to explore the functions of the three transcription factors belonging to the VND cluster, we checked their expression levels during strawberry fruit development in “Yellow Wonder” (Figure 1A). Compared with *FvNST1b*, the expressions of *FvVND4a*, *FvVND4b* and *FvVND4c* were relatively low at the earlier stages. The expression of *FvVND4c* was higher than those of other members in this group. The *FvVND4c* expression was highest at the S7 stage, when the firmness of fruits reached the maximum, suggesting its involvement in the subsequent ripening transition. To examine their subcellular localization in vivo, *FvVND4s* were fused between a C-terminal GFP tag and the CaMV 35S promoter (Figure 1B). The transient expression of *FvVND4s* in strawberry fruit demonstrated that FvVND4b and FvVND4c are exclusively located in the nucleus, while FvVND4a is located in both the cell membrane and nucleus. Hence, the results of the subcellular localization of FvVND4s were consistent with their functions as transcription factors.

To examine the expression of *FvVND4s* during vascular differentiation, we established an in vitro cultural system to induce vascular xylem cells using *F. vesca* leaf discs by modifying the Vascular Cell Induction Culture System Using Arabidopsis Leaves (VISUAL) [30,31]. The system successfully differentiated mesophyll cells into xylem cells with SCW thickening (Supplemental Figure S1A,B). We examined the expression level of *FvVND4s* during the culture process, and showed that *FvVND4a* and *FvVND4c* were significantly increased during the induction process (Supplemental Figure S1C). These results suggest that the expression change of *FvVND4s* is associated with xylem induction and SCW thickening in *Fragaria vesca*.

### 2.2. Overexpression of FvVND4c Induces SCW Formation and Color Changes

**Figure 2:**
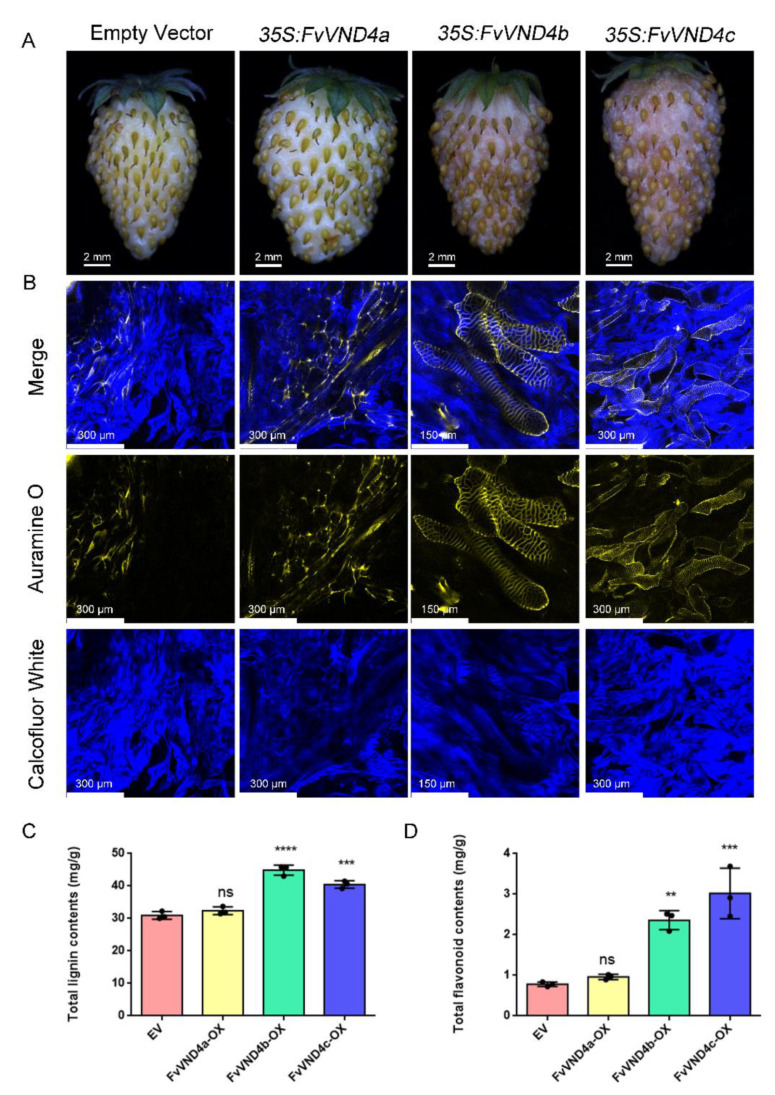
FvVND4c positively regulates SCW formation and flavonoid accumulation. (**A**) Images of fruits 7days after infiltration of agrobacterium with *FvVND4-GFPs* overexpression vectors, or empty vectors. (**B**) Images of agrobacterium-infected fruit sections stained with Calcofluor White and Auramine O. The images of the Calcofluor White channel and the Auramine O channel and the merged images are shown. Total lignin contents (**C**) and flavonoid contents (**D**) were measured in fruits after *FvVND4-GFPs* overexpression. Error bars represent the SD of three independent replicates, asterisks indicate significant *p*-values determined by the *t*-test (**, *p* < 0.01; ***, *p* < 0.001; ****, *p* < 0.0001). EV, empty vector. OX, overexpression. ns: not significant.

To investigate the functions of FvVND4s, transient overexpressions of *FvVND4s* were performed in “Yellow Wonder” fruit at 20 days after pollination—the transitional phase from green to white [32]. Seven days after infiltration, strawberry fruits overexpressing *FvVND4b* or *FvVND4c* changed their color from white to dim red, while *FvVND4a* overexpression had no effect on the fruit color (Figure 2A). To examine the formation of SCW, sections of strawberry fruits were stained with Calcofluor White (staining all cell walls) and Auramine O (staining only SCW) [33]. As shown in Figure 2B, the overexpression of *FvVND4b* or *FvVND4c* but not *FvVND4a* induced SCW thickening. Similar results were obtained in relation to transient overexpression in tobacco leaves (Supplemental Figure S2A), indicating that *FvVND4b* and *FvVND4c* influences SCW thickening.

Lignin and flavonoids are indispensable components in the development of cell wall and fruit color in strawberries, respectively [34]. Since the fruit color changed and the SCW thickened following *FvVND4c* overexpression, we checked the total lignin and flavonoids contents of the fruit tissues after seven days of *FvVND4s* overexpression. Both total lignin contents and flavonoid contents were increased upon *FvVND4b* and *FvVND4c* overexpression, and remained unchanged upon the overexpression of *FvVND4a* (Figure 2C,D). Focusing on genes related to vascular elements and flavonoid biosynthesis, the overexpression of *FvVND4c* but not *FvVND4a* significantly altered their expression (Supplemental Figure S2B). These data are consistent with the idea that *FvVND4c* but not *FvVND4a* induces ectopic SCW formation and fruit color changes by regulating the genes involved in lignin and flavonoid biosynthesis. Thus, we chose *FvVND4c* for further exploration.

**Figure 3:**
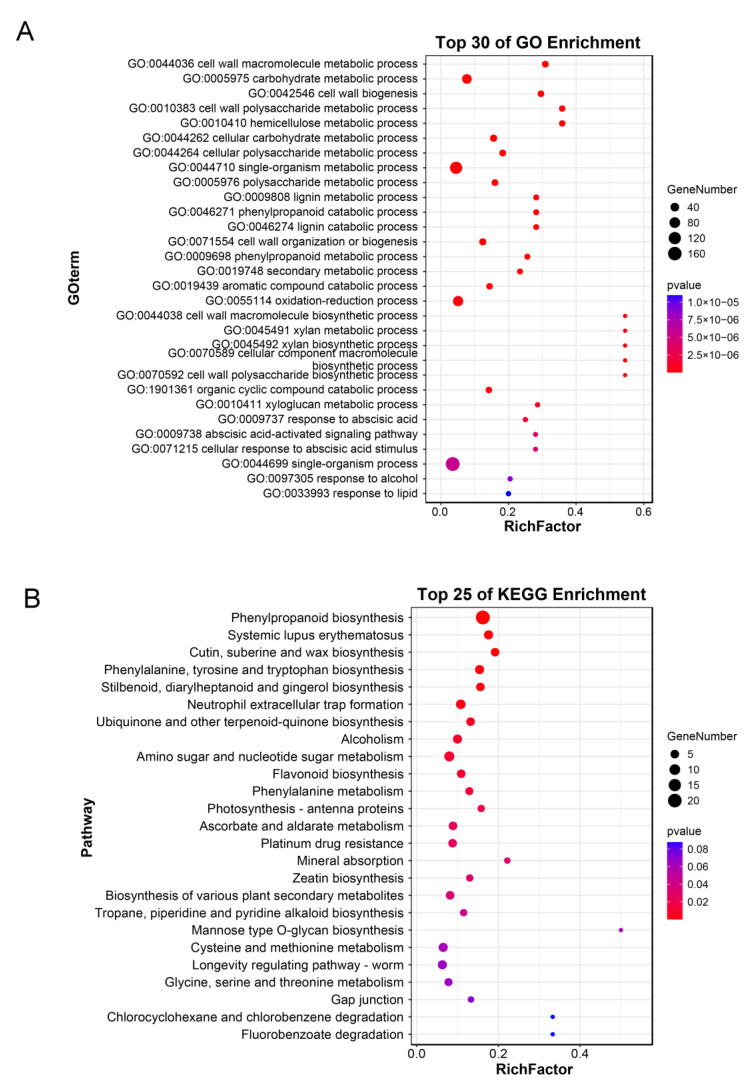
Functions of the differentially expressed genes upon *FvVND4c* overexpression. (**A**) GO enrichment analysis and (**B**) KEGG enrichment analysis. The RNA-seq libraries of *F. vesca* fruits for the comparative analyses were respectively transformed with *35S:FvVND4c* and the empty vectors. Terms with *p* < 0.05 and |log_2_(Fold change)| > 1.0 are shown. Size of the circles represent gene numbers, while the colors of the circles represent *p*-values.

We further explored how the whole transcriptome responds to the overexpression of *FvVND4c*. Comparing RNA-seq libraries from transformations of *FvVND4c* and the empty vectors, a total of 690 differential expression genes were found. Specifically, 584 genes were upregulated and 106 genes were downregulated (Supplemental Table S1). GO analysis showed that these genes were enriched via the regulation of SCW biogenesis, xylan, lignin, and hemicellulose biosynthetic processes (Figure 3A). The pathway enrichment analysis (KEGG) showed that they were involved in the regulation of phenylpropanoid and flavonoid biosynthesis pathways (Figure 3B). Taken together, these results suggest that the overexpression of *FvVND4c* induces SCW formation and color changes through the regulation of genes involved in lignin and flavonoid biosynthesis.

### 2.3. FvVND4c Activates Expression of FvMYB46 and FvMYB83

**Figure 4:**
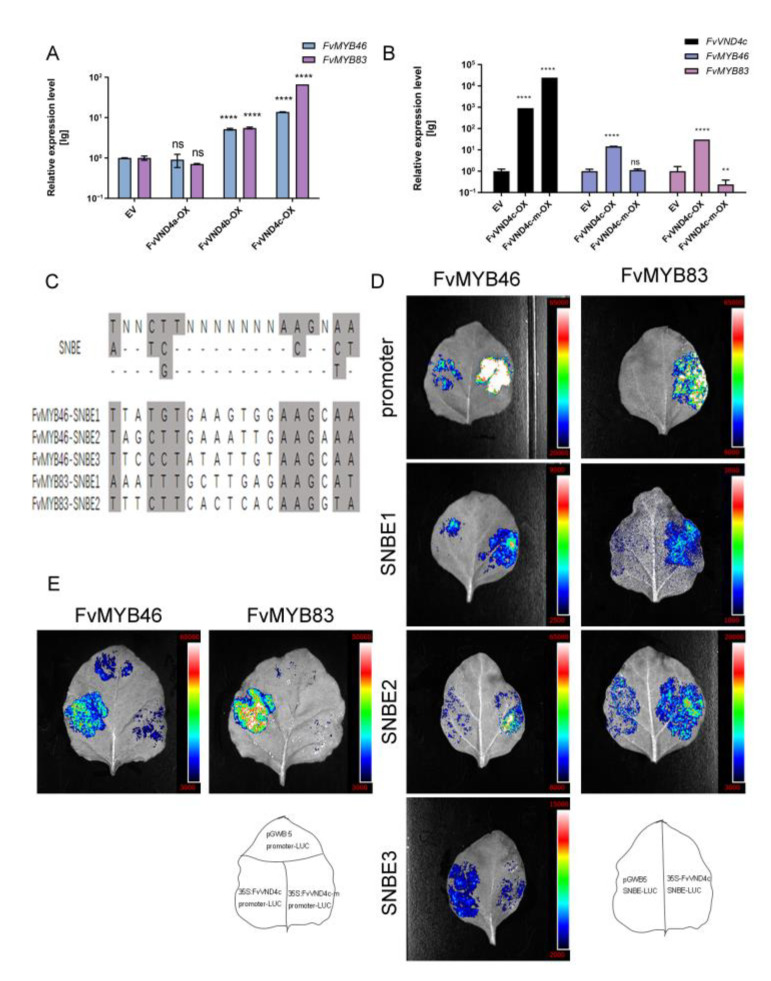
Expressions of *FvMYB46* and *FvMYB83* were upregulated by FvVND4c. (**A**,**B**) Q-PCR analysis of transcript levels for *FvMYB46* and *FvMYB83* in strawberry fruits 7 days after overexpression of *FvVND4s* (**A**) or *FvVND4c-m* (**B**). Error bars represent SD of three independent replicates, asterisks indicate significant *p*-values determined by the *t*-test (**, *p* < 0.01; ****, *p* < 0.0001). EV, empty vector. OX, overexpression. (**C**) Sequences of the SNBE elements in promoters of *FvMYB46* and *FvMYB83*. (**D**,**E**) Transactivation analysis of *FvVND4c* (**D**) and *FvVND4c-m* (**E**) for the full promoters of *FvMYB46* and *FvMYB83*, and for SNBE elements with the LUC reporter system. The whole 1.5 kb promoter sequences or three tandem repeats of SNBE sequence from promoter sequences were fused with the minimal CaMV 35S promoter (m35S) to drive the LUC reporter gene. The reporter construct was co-transformed with the effector of 35S: *FvVND4c* (**D**) or 35S: *FvVND4c-m* (**E**) into tobacco leaves for transactivation analysis. Note that in (**D**), except for SNBE3, the LUC signals from the co-transformation of the 35S: *FvVND4c* effector and reporter (right) were much stronger than those from the transformation of the reporter construct alone (left); in (**E**), the LUC signals from the co-transformation of the 35S: *FvVND4c* effector and reporter (left) were much stronger than those from the transformation of the 35S: *FvVND4c-m* effector and reporter (right), or the reporter construct alone (up). ns: not significant.

In order to further elucidate how FvVND4c induced ectopic lignification and fruit color change, we next explored its potential downstream genes. AtMYB46 and AtMYB83 are two downstream factors of NACs that acted as second-layer master switches in SCW formation in Arabidopsis [12,13]. Their homologs in strawberry, *FvMYB46* and *FvMYB83*, also showed increased expression levels upon *FvVND4c* overexpression, as confirmed by both the transcriptomic data (Supplemental Table S1) and the qRT-PCR measurements (Figure 4A). In contrast, the overexpression of *FvVND4a* had no effects (Figure 4A). Next, we constructed a mutant version of FvVND4c that lacks the DNA binding site (FvVND4c-m) [35]. The overexpression of *FvVND4c-m* did not induce SCW thickening or color changes after ectopic expression in strawberry fruit (Supplemental Figure S3A). On the molecular level, the overexpression of *FvVND4c-m* had no effect on *FvMYB46* expression, and decreased the expression of *FvMYB83* (Figure 4B). These results suggest that, similar to Arabidopsis, *FvMYB46* and *FvMYB83* are downstream genes of FvVND4c in *F. vesca*.

Previous studies revealed that SCW-related NACs directly activate the target genes participating in SCW formation by binding to the SNBE element, a palindromic 19-bp consensus sequence, (T/A) NN (C/T) (T/C/G) TNNNNNNNA (A/C) GN (A/C/T) (A/T) [36]. Several SNBE sites are present in the 1.5 kb promoter sequences of *FvMYB46* and *FvMYB83* (Figure 4C). In order to test whether FvVND4c regulates *FvMYB46* and *FvMYB83* through the SNBE elements, we performed a luciferase reporter assay in tobacco leaves (Figure 4D,E). Transient expression assays revealed that the overexpression of *FvVND4c* induced reporter expression for the whole promoters, and the SNBE1 and SNBE2 sites of *FvMYB46* and *FvMYB83* (Figure 4D). However, the overexpression of *FvVND4c-m* did not activate the promoters (Figure 4E). These results imply that FvVND4c positively regulates the expression of *FvMYB46* and *FvMYB83,* likely through direct binding to the SNBE sites.

### 2.4. Overexpression of FvMYB46 Induces SCW Thickening and Flavonoid Accumulation

**Figure 5:**
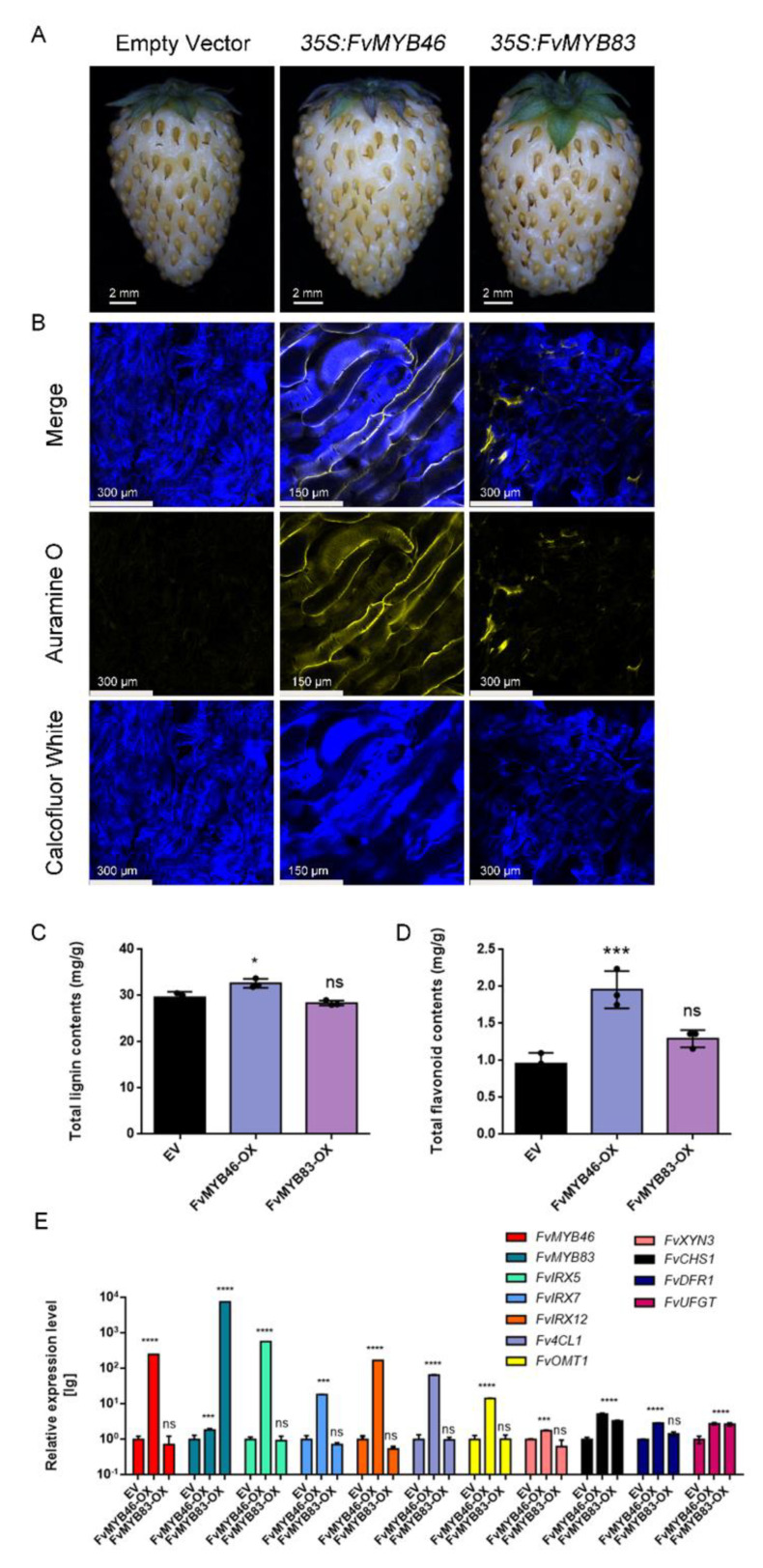
FvMYB46 positively regulates SCW formation and flavonoid accumulation. (**A**) Phenotypes of fruits agro-infiltrated with *FvMYB46* and *FvMYB83* overexpression vector, or empty vector. Fruits were imaged 7 days after agrobacterium infiltration. (**B**) Images of hand-sectioned fruits stained with Calcofluor White and Auramine O. (**C**,**D**) Measurements of total lignin contents (**C**) and flavonoid contents (**D**) in fruits infiltrated with agrobacterium containing the *FvMYB46* or *FvMYB83* overexpression vector. (**E**) Q-PCR analysis of transcript levels of SCW-related genes (*FvIRX5*, *FvIRX7*, *FvIRX12*, *Fv4CL1*, *FvOMT1*, *FvXYN3*) and flavonoid biosynthesis-related genes (*FvCHS1*, *FvDFR1*, *FvUFGT*) in fruits overexpressing *FvMYB46* or *FvMYB83*. In (**C**–**E**), the error bars represent SD values of three independent replicates, asterisks indicate *t*-test *p*-values for significant differences (*, *p* < 0.05; ***, *p* < 0.001; ****, *p* < 0.0001). EV, empty vector. OX, overexpression. ns: not significant.

The results described above suggest that FvMYB46 and FvMYB83 are FvVND4c-activated transcriptional regulators. To examine their involvement in SCW biosynthesis and flavonoid accumulation, we overexpressed full-length *FvMYB46* and *FvMYB83* to observe their physiological consequences. Seven days after agrobacterium transformation, fruits overexpressed with *FvMYB46* exhibited no color changes, but showed induced SCW thickening. No effects were observed upon *FvMYB83* (Figure 5A,B). Consistently, the total lignin and flavonoids contents increased only upon *FvMYB46* overexpression (Figure 5C,D). The genes related to SCW thickening and flavonoid biosynthesis that were upregulated upon *FvVND4c* overexpression (Supplemental Figure S2B) can also be upregulated by *FvMYB46* overexpression, but not by *FvMYB83* (Figure 5E). These results indicate that FvMYB46 is downstream of FvVND4c and induces SCW thickening and flavonoid accumulation.

### 2.5. FvVND4c and FvMYB46 Co-Regulate Genes of SCW Thickening and Flavonoid Accumulation

**Figure 6:**
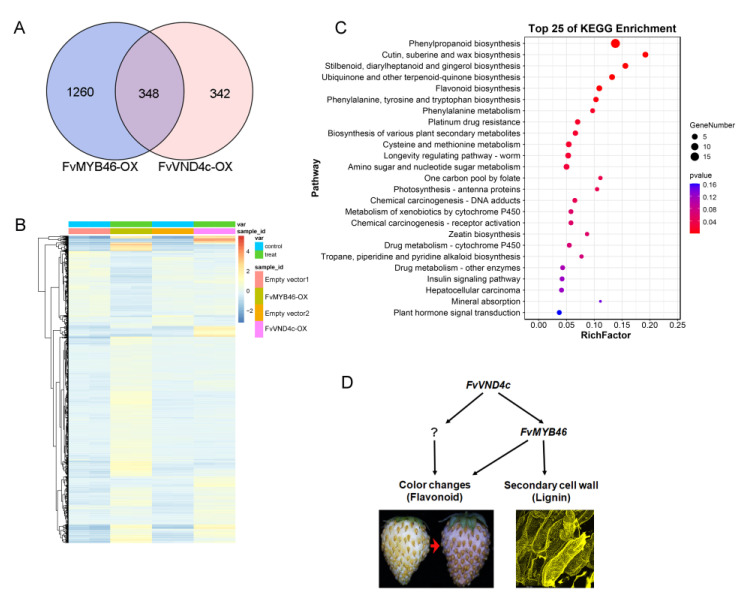
Transcriptome analysis on differentially expression genes induced by transient overexpression of *FvVND4c* or *FvMYB46* in strawberry fruits. (**A**) Venn diagram displaying the differential expressions of genes induced by *FvVND4c* and by *FvMYB46* transient overexpression. (**B**) Heatmap of the genes differentially expressed in response to *FvVND4c* or *FvMYB46* transient overexpression. (**C**) KEGG enrichment analysis of the 384 genes differentially expressed in response to both FvVND4c and FvMYB46 transient overexpression. (*p* < 0.05, |log_2_(Fold change) > 1.0|). Sizes of the circles represent gene numbers, and the colors encode the *p*-values. (**D**) The model of the *FvVND4c* regulatory network.

We next explored the targets downstream of *FvMYB46*. A total of 1608 genes were differentially expressed between strawberry fruits respectively infiltrated with agrobacterium containing *35S:FvMYB46* and CaMV 35S vectors. Of these genes, 1172 were upregulated and 436 were downregulated (Supplemental Table S2). GO analysis of all differential expression genes induced by FvMYB46 has enriched the terms related to regulation of cell wall biosynthetic processes (Supplemental Figure S4A). Among the differentially expressed genes induced by FvVND4c, 348 genes were also differently expressed when overexpressing FvMYB46 (Figure 6A,B), thereby representing the common downstream genes of the two TFs. KEGG enrichment analysis based on the common downstream genes showed that they were involved in the regulation of phenylpropanoid and flavonoid biosynthesis pathways (Figure 6C). Genes in these pathways all contribute to lignin and flavonoid synthesis (Supplemental Figure S4B). These results support the idea that FvVND4c regulates FvMYB46 to promote secondary cell wall thickening and flavonoid accumulation.

## 3. Discussion

The strawberry fruit ripening process is an important research area, as it directly affects commercial values. The process proceeds via the coordination of multiple signaling processes, which lead to changes in chemical composition. To date, multiple transcription factors have been revealed to play roles in the process [37,38,39]. The ripening process of strawberry fruit is accompanied by softening and cell wall degradation [40,41]. SCW thickening provides mechanical support for various plant tissues, and contributes to fruit firmness [42,43]. NAC (NAM, ATAF, and CUC) TFs constitute a large protein family that plays important regulatory roles in plant development and environmental responses [44]. Several NAC TFs are the master switches that turn on the entire SCW biosynthetic program [15,42,45]. NAC TFs are also involved in the regulation of ripening-associated processes in fruits [23,46,47]. Our previous study has shown that FvNST1b is an activator of SCW thickening [29]. In this study, we explored the function of FvVND4s, that are closely related to FvNST1b [29]. We found that FvVND4c but not FvVND4a induces SCW thickening and color changes in strawberry fruit. FvVND4c cooperated with FvMYB46 through regulating the phenylpropanoid pathway. Their downstream targets involve cell wall formation-related TFs and flavonoid biosynthesis-related TFs, and therefore lead to alterations in total lignin and flavonoid contents.

The strawberry fruit developmental process has previously been divided into 12 stages. The early phase, including seven stages S1–S7, is characterized by a gradual increase in fruit (receptacle and achene) size, weight, and firmness. The later phases are the ripening phases, divided into RS1 to RS5 [32]. Our quantitative gene expression analysis performed throughout fruit development has shown that the expression of *FvVND4c* was highest in S7 stage, when the firmness of fruits is the hardest. A previous report showed that there is a sudden drop in the firmness between S7 and RS1, when the expression level of *FvVND4c* decreases (Figure 1A) [32]. Therefore, in the S7 stage, FvVND4c may play a role in maintaining firmness. This hypothesis is also supported by the observation that the overexpression of *FvVND4c* induced SCW thickening and lignin accumulation (Figure 2B,C), which increased the firmness [23,48]. On the other hands, fruits undergo a transition to ripening at S7 [32], and strawberry ripening leads to an increase in the contents of sugars, anthocyanins, volatile compounds, and vitamins [23]. Indeed, our transcriptome analyses indicate that FvVND4c is involved in the regulation of phenylpropanoid and flavonoid biosynthesis pathways (Figure 3A,B), which can cause color changes and increase flavonoid contents (Figure 2A,D). Taken together, FvVND4c may play a key role in promoting fruit firmness and color changes by regulating SCW thickening, the total lignin content, and the total flavonoids content, respectively.

In the network regulating the SCW thickening in Arabidopsis, NACs are the first layer of master switches [6,7,8]. AtMYB46 and AtMYB83 are present downstream of NACs, and act as second-layer master switches [12,13]. In this work, we also characterized FvMYB46 and FvMYB83, the downstream candidate factors of FvVND4c. Transcriptome analysis showed that *FvMYB46* and *FvMYB83* are differentially expressed after *FvVND4c* overexpression (Supplemental Table S1). However, the overexpression of *FvMYB46* did not induce fruit color changes but promoted ectopic SCW thickening, and *FvMYB83* overexpression had little effect (Figure 5A,B). Although FvMYB46 did not induce fruit color changes, it increases the total flavonoids content and the expression level of flavonoid biosynthesis-related genes (Figure 5D,E). Previous studies have shown that AtVNDs and AtMYB46 regulate SCW thickening and lignin synthesis [10,49,50,51], but their contributions to flavonoid synthesis have not been analyzed in detail yet. The biosynthetic pathways for lignin and flavonoids share common precursors from the general phenylpropanoid pathway [34]. Our transcriptome analyses indicate that FvVND4c and FvMYB46 positively regulate the phenylpropanoid biosynthesis pathways that contribute to lignin and flavonoid synthesis (Figure 6C, Supplemental Figure S4B). In winter jujube, *F5H* expression is promoted by an NAC (LOC10743523), which leads to lignin biosynthesis during fruit ripening. The pigmentation of fruit is also regulated by NAC proteins, as in the case of this report. Moreover, the MYB activator (LOC107425254) and the MYB repressor (LOC107415078) also regulate *CCR* and *F5H* to control lignin biosynthesis and induce cell wall lignification, further suggesting the existence of similar molecular mechanisms to those found in this report [52]. While overexpressing either *FvVND4c* or *FvMYB46* caused cell wall lignification and flavonoid accumulation, only *FvVND4c* (and not *FvMYB46*) induced fruit color changes, suggesting that enzymes converting flavonoids to coloring pigments are regulated only by FvVND4c (Figure 6D). Future efforts will focus on identifying these enzymes and elucidating their regulation mechanisms.

Anthocyanin is a main component leading fruit color change in *Fragaria genus* [53]. Several transcription factors were shown to regulate genes of anthocyanin biosynthesis. For example, FvMYB10 in *F. vesca* and FaMYB10 in *F. ananassa* positively regulate anthocyanin biosynthesis [28,54]. Comparing the genomes of red and white *F. vesca* accessions has identified a candidate SNP in *FvMYB10* that determines the fruit color [28]. The overexpression of *FvMYB10* from the red fruit “Ruegen” in the fruit of “Yellow Wonder” caused red pigmentation at the injection sites [28]. *35S:FvMYB10* lines of “Alpine” strawberry *F. vesca ssp. vesca* were also pigmented, and mature fruit from these lines had dark red/purple skin and red flesh, compared with the red skin and white flesh found in wild-type mature fruit. The levels of transcripts encoding anthocyanin biosynthetic genes *FvCHS*, *FvF3H*, *FvDFR*, *FvLDOX*, and *FvUFGT* showed elevations in all *35S:FvMYB10* lines and reductions in two *FvMYB10 RNAi* lines, compared with wild-type controls [55]. In this work, we found that FvVND4c can also induce the expression of *FvCHS*, *FvDFR* and *FvUFGT*, but not *FvF3H* or *FvLDOX* (Supplemental Figure S2C). As a potential FvVND4c downstream gene, FvMYB46 also promotes the expression of *FvCHS*, *FvDFR* and *FvUFGT* (Figure 5E). We found that the expression of *FvMYB10* did not change significantly following *FvVND4c* overexpression. Therefore, FvVND4c and FvMYB10 may co-regulate *FvCHS*, *FvDFR* and *FvUFGT* through different pathways, which together lead to fruit color changes.

As transcriptional regulation is complex, some TFs work as both positive regulators and negative regulators in a context-dependent manner. Some transcription factors such as PtMYB8 in *Pinus taeda* and AtMYB20 in *Arabidopsis thaliana* negatively regulate the biosynthesis of flavonoids, but increase the contents of lignin in the plant [56,57]. However, other transcription factors promote the synthesis of flavonoids at the cost of inhibiting lignin synthesis, such as AtMYB75, AtMYB90 and PtMYB6 in poplar [58,59,60]. There are also transcription factors that positively regulate both pathways, such as EgMYB88 in *Eucalyptus grandis*. The overexpression of *EgMYB88* caused a substantial increase in the levels of both flavonoids and lignin [61]. Similar to EgMYB88, our data show that the ectopic expression of *FvVND4c* and *FvMYB46* positively regulated both SCW thickening (lignin synthesis) and flavonoid accumulation. Therefore, this work reveals novel dual-pathway activating factors. Previous findings combined with our results illustrate an abundant reservoir of strategies by which plant can direct their carbon flow towards the syntheses of flavonoids and lignin [34].

In summary, we find that the NAC TF FvVND4c is highly expressed in the fruit ripening transition stage, and localizes in the nucleus. FvVND4c is able to induce the ectopic deposition of SCW and fruit color changes in *Fragaria vesca*, and increase the total lignin content and flavonoid content. FvMYB46, a potential downstream factor of FvVND4c, has similar effects to FvVND4c, but fails to induce the color change. An analysis of the promoter of *FvMYB46* also suggested that FvVND4c positively regulates SCW thickening and flavonoid accumulation through FvMYB46. These findings will advance our understanding regarding the regulatory network during the development and ripening of strawberry fruit.

## 4. Materials and Methods

### 4.1. Plant Material and Growth Conditions

Diploid strawberry plants (*Fragaria vesca*) of the type “Yellow Wonder” 5AF7 (YW5AF7) [27], planted in pots (90 mm × 90 mm × 90 mm), were used in this study. The seedlings were grown and maintained in a growth room with the following conditions: 22 °C, 60% humidity, and a 16 h photoperiod. Hand pollination was performed using downy water bird feathers to obtain pollinated fruit. The samples used for RNA isolation were frozen in liquid nitrogen immediately after collection, and then stored at −80 °C.

### 4.2. Plasmid Construction

The primers used for plasmid construction are listed in Supplemental Table S3. *FvVND4s* were amplified from the DNA of “Yellow Wonder” strawberry using Primer STAR^®^ GXL DNA Polymerase (TaKaRa, Maebashi, Japan), subcloned into pDONR221, and then inserted into the binary vector pGWB5 using Gateway^®^ Technology. The coding regions of the *FvMYB46* and *FvMYB83* genes were amplified from the cDNA of the “Yellow Wonder” strawberry using Primer STAR^®^ GXL DNA Polymerase (TaKaRa, Maebashi, Japan), subcloned into pDONR221, and then inserted into the binary vector pGWB5 using Gateway^®^ Technology. The correct fusion constructs were transferred into the *Agrobacterium tumefaciens* strain GV3101 by the freeze–thaw method.

### 4.3. RNA Extraction and Q-PCR Analysis

Total RNA from the strawberry samples was extracted using the polysaccharide and polyphenolics-rich RNAprep Pure Kit (Tiangen, Beijing, China), while RNA quality was detected by NanoDrop. Samples with RNA concentrations greater than 100 ng/uL and 260/280 values between 1.9 and 2.1 were qualified. We took 1 ng RNA for reverse transcription. The cDNA used for quantitative reverse transcription–PCR (q-PCR) analysis was synthesized using one-step genomic DNA removal and a cDNA synthesis kit (Tiangen, Beijing, China). The cDNA samples were diluted 1:10 with water; 5 µL of the diluted cDNA was used as a template for Q-PCR. Q-PCR was performed in the ABI 7500 Real-Time PCR System (Applied Biosystems, Waltham, MA, USA) using the RealStar Fast SYBR qPCR Mix (GenStar, Beijing, China). The primers are shown in Supplemental Table S3; the Actin gene used in previous publications was used as the internal control [32]. The PCR program included an initial denaturation step at 95 °C for 3 min, followed by 40 cycles of 10 s at 95 °C, and 30 s at 60 °C. The analysis was performed using three biological samples and three technical repeats. The relative expression levels of target genes were calculated with the formula 2−ΔΔct.

### 4.4. Transient Transformation of Strawberry Fruit and Subcellular Localization Analysis

Transient transformation of the strawberry fruits was carried out using agroinfiltration as previously described [62]. GV3101 strains, which harbor FvVND4s overexpression vectors or a control vector, were resuspended in infection buffer and shaken for 2 h at 28 °C, and infiltrated into the “Yellow Wonder” fruit flesh at 20 d after pollination using syringes of 1 mL capacity. The needle tip was inserted into the fruit center from the top, and then the Agrobacterium suspension was slowly and evenly injected into the fruits until the strawberry fruit was completely infected. After the infection, the fruits were incubated under the conditions required for the different experimental aims. At 72 h post-infiltration, the GFP signal was visualized using a confocal fluorescence microscope (Leica Confocal microscope SP8X; Leica Microsystems GmbH, Wetzlar, Germany) with a 10× objective lens, a 488 nm tunable white light laser for excitation, and a 499 to 551 nm bandwidth for detection. At 7 days post-infiltration, images were taken, and tissues were collected for downstream analysis.

### 4.5. Transient Expression Assays in Tobacco (Nicotiana benthamiana) Leaves

The coding regions of *FvVND4s*, 3 kb promoters of *FvMYB46* and *FvMYB83*, and SNBEs were amplified and cloned into the effector (35S-transcription factor) and reporter (SNBE-mini35S-luciferase) vectors, respectively. The reporter and effector constructs were transformed into the *A. tumefaciens* strain GV3101 and injected into tobacco leaves. At three days after infiltration, LUC activity was observed with a cold CCD camera (Tanon-5200). Cotton swabs were used to evenly apply 1 mM luciferin substrate (Diluted with 1% Triron-100) (YEASEN, Shanghai, China) on both sides of the tobacco leaves. Images were taken after 10 min of substrate darkening, when the instrument had cooled to below −30 °C.

### 4.6. Measuring Total Lignin Contents of Fruits

At 7 days after infiltration, the infected strawberry fruits were sampled and dehydrated at 65 °C; we then removed the achenes and ground them into powder for later use. The extraction and measurement of the total lignin contents were performed according to the instructions of the Lignin Content Kit (Acetylation Method) (G0708W) (Grace Biotechnology, Suzhou, China). All samples contained three biological replicates.

### 4.7. Measuring Total Flavonoid Contents of Fruits

At 7 days after infiltration, the infected strawberry fruits were sampled, and we removed the achenes for later use. The extraction and measurement processes of total flavonoid contents were performed according to the protocol of the Total Flavonoid Content Kit (G0118W) (Grace Biotechnology, Suzhou, China). All samples contained three biological replicates.

### 4.8. Fruit Sectioning and Staining

At 7 days after infiltration, the infected strawberry fruits were embedded in 10% agarose gel, and cut into 200 µm-thick sections with a vibratome (Leica VT1000 S). Strawberry fruit sections were fixed with 4% PFA for 60–120 min at 23–25 °C with vacuum treatment. After fixation, the materials were washed twice for 1 min in 1 × PBS and moved to the clearing solution. After rinsing in 1 × PBS, the plant material was transferred into the ClearSee solution [33] and cleared overnight at room temperature. We prepared 0.1% Auramine O in ClearSee solution, and the materials were stained overnight. Then, the materials were washed for at least 1 h with gentle shaking. The materials were transferred to 0.1% Calcofluor White in ClearSee solution and stained for 30 min; the materials were then washed in ClearSee for 30 min with gentle shaking. The materials were analyzed with a Leica TCS SP8X inverted confocal microscope. Imaging using Calcofluor White was performed with a 405 nm diode laser for excitation, and the detection bandwidth was 425–475 nm. Imaging with Auramine O was performed using a 488 nm bandwidth supplied by a tunable white light laser, and the bandwidth was detected at 505–530 nm.

### 4.9. In Vitro Induction Culture of Vascular Cells of Fragaria vesca

At ages of 3–4 weeks, the third and fourth leaves of the strawberry plants were isolated, and leaf discs with sizes of 1 mm were cultured in MS liquid medium containing hormones (Bikinin 20 µM, 2,4-D 5 mg·L^−1^, Kinetin 1 mg·L^−1^). The leaf discs with medium were placed on the petri dishes and rotated on a shaker with a rotating speed of 110 rpm at 22 °C, under continuous white light. Mesophyll cells were differentiated into xylem cells from day 7.

### 4.10. Transcriptome Analysis

Seven days after injection, fruits infiltrated with agrobacterium containing either empty vector, *35S:FvVND4c*, or *35S:FvMYB46* were sampled for RNA-seq analysis. After the total RNAs were isolated and tested as described (interrupting RNA using the NEB Next^®^ Ultra^TM^ II RNA Library Prep Kit for Illumina^®^), sequencing was performed on the Illumina NovaSeq 6000 platform. A 6G sequencing depth was employed for each sample. After preprocessing the RNA-seq data with fastp v.0.20.1 [63], the reads were mapped to the *Fragaria vesca* genome (https://www.rosaceae.org/species/fragaria_vesca/genome_v4.0.a2, accessed on 17 November 2022) using hisat2 v.2.2.1 [64] with default parameters. An average of 45.6 million paired-end 150 bp raw reads per sample were obtained, and around 90.6% of the clean reads (41.3 million) were mapped to the *Fragaria vesca* reference genome. The genome coverage was around 28.1, the calculation formula for which is: $\mathrm{Genome}\;\mathrm{Coverage}=\frac{\mathrm{Clean}\;\mathrm{Reads}*\mathrm{Read}\;\mathrm{Length}}{\mathrm{Total}\;\mathrm{Genome}\;\mathrm{Length}}$. Samtools v.1.9 [65] was used to tidy the alignment results, and to remove the duplicated sequences derived from PCR. Finally, the number of matched reads was determined using FeatureCounts v.2.0.3 [66] and imported into R statistical software (4.2.2), wherein the differential expression analysis was accomplished using the DESeq2 with a cut-off of *p*-value (0.05) and a cut-off of absolute log2 fold change (1). Gene Ontology (GO) and pathway enrichment (KEGG) analyses of the genes that were differentially expressed in the biological process were conducted using the OmicShare software (https://www.omicshare.com/tools/, accessed on 24 November 2022). All samples contained three biological replicates. All data including the completed metadata worksheet, raw data and processed data have been submitted to the GEO database, under the accession number GSE225542.

## Data Availability

Raw data for transcriptome analysis were deposited into the Gene Expression Omnibus database under accession number GSE225542 and are available at the following URL: https://www.ncbi.nlm.nih.gov/geo/query/acc.cgi?acc=GSE225542.

## Supplementary Materials

The following supporting information can be downloaded at: https://www.mdpi.com/article/10.3390/ijms24098110/s1.
