# Supplementary material for: Ectopic Expression of FvVND4c Promotes Secondary Cell Wall Thickening and Flavonoid Accumulation in Fragaria vesca
Source: Int J Mol Sci. 2023 Apr 30;24(9):8110. doi: 10.3390/ijms24098110 (PMC10179399; doi:10.3390/ijms24098110)
Supplement: Supplementary file 1 [file ijms-24-08110-s001.zip › ijms-2351640-supplementary.pdf]

Supplemental Figure S1.

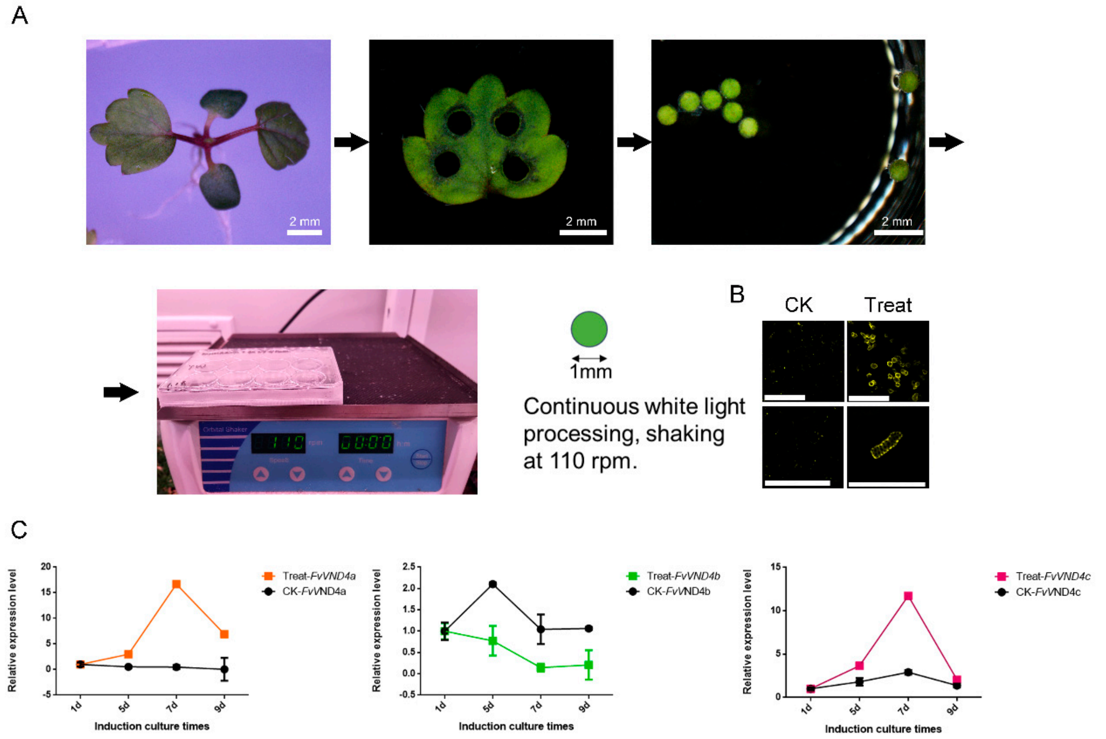

Supplemental Figure S1. In vitro induction culture system of vascular cells in *Fragaria vesca* leaves.

(A) Sampling and culture procedures of strawberry leaf discs. The third and fourth leaves of the strawberry plants at the age of 3-4 weeks were isolated and the leaf discs with the size of 1 mm were cultured in MS liquid medium containing hormones (Bikinin 20 μM, 2,4-D 5 mg·L^{-1}, Kinetin 1 mg·L^{-1}). The petri dishes were placed on a shaker with a rotating speed of 110 rpm, at 22℃, and treated with continuous white light.

(B) Images of xylem cells isolated from strawberry leaf disks and stained by Auramine O after induction culture. Left two graphs from the control group that cultured with only MS liquid medium, right two graph form the treatment group that cultured with MS liquid medium containing hormones (Bikinin 20 μM, 2,4-D 5 mg·L^{-1}, Kinetin 1 mg·L^{-1}). Mesophyll cells began to transform into cells with secondary cell wall thickening on the 7th day, and the conversion rate reached the highest on the 9th day. Bar=100 μm.

(C) Q-PCR analysis of expression level for *FvVND4s* during the induction culture process.

Supplemental Figure S2.

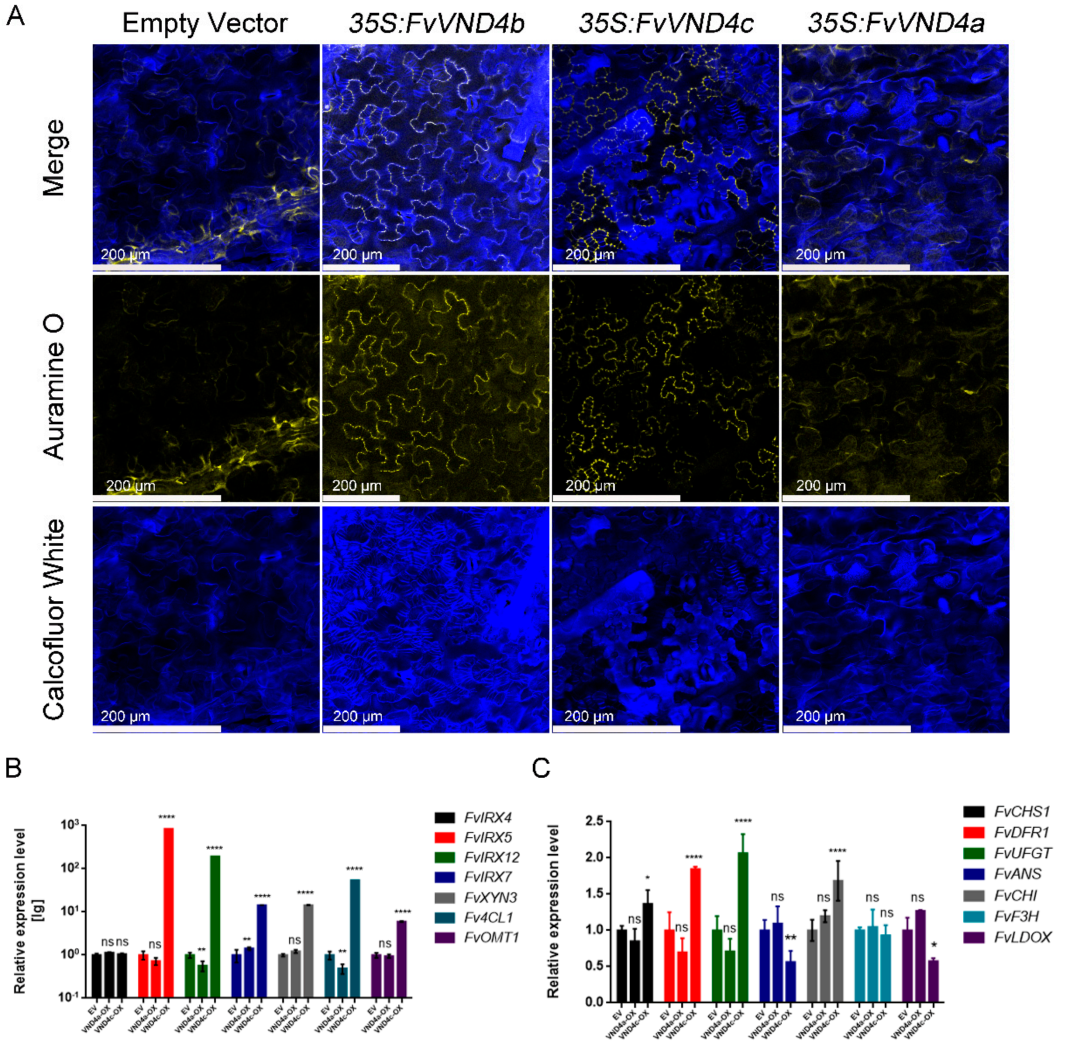

Supplemental Figure S2. Overexpression of *FvVND4s* induces secondary cell wall thickening and flavonoid biosynthesis genes

(A) Images of tobacco mesophyll cells infected by agrobacterium containing either empty vector or *35S: FvVND4s* stained with Calcofluor White and Auramine O.

(B-C) Q-PCR analysis of transcript levels of secondary cell wall formation (B) and flavonoid biosynthesis (C) related genes 7 days after overexpression of FvVND4a and FvVND4c in strawberry. Error bars represent SD of three independent replicates, asterisk indicates values that were determined by the t-test to be significantly different from the control (*, $p < 0.05$; **, $p < 0.01$; ***, $p < 0.001$; ****, $p < 0.0001$).

Supplemental Figure S3.

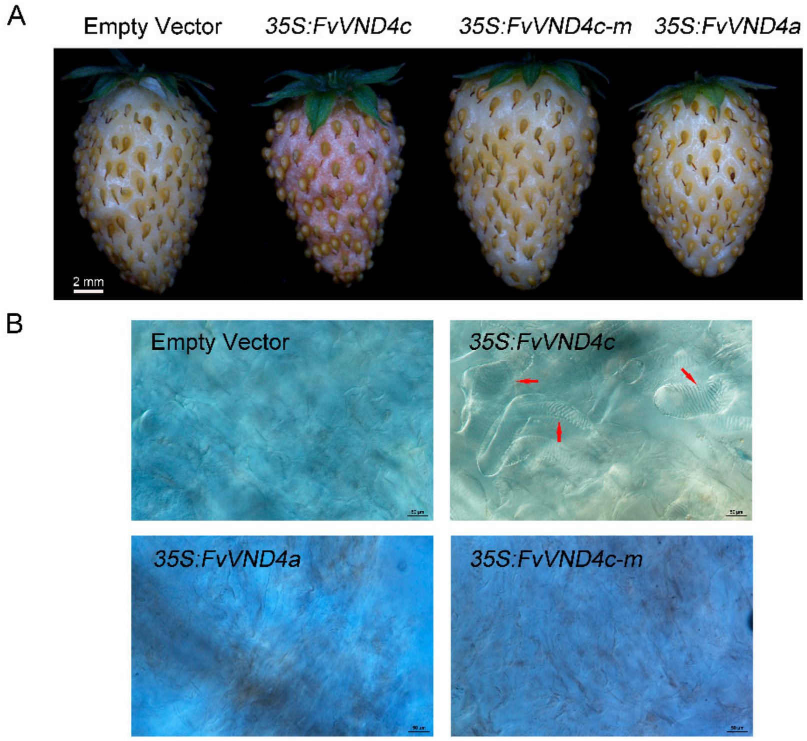

Supplemental Figure S3. Assessment of mutation in FvVND4c

(A) Phenotypes of fruits that were infiltrated with agrobacterium containing FvVND4c and FvVND4c-m overexpression and empty vector at 7 days.

(B) DIC images of hand-sectioned infected fruits infected by agrobacterium containing either empty vector, *35S: FvVND4c, 35S:VND4a* or *35S: FvVND4c-m.* Arrows show TE-like cells induced by FvVND4c overexpression.

Supplemental Figure S4.

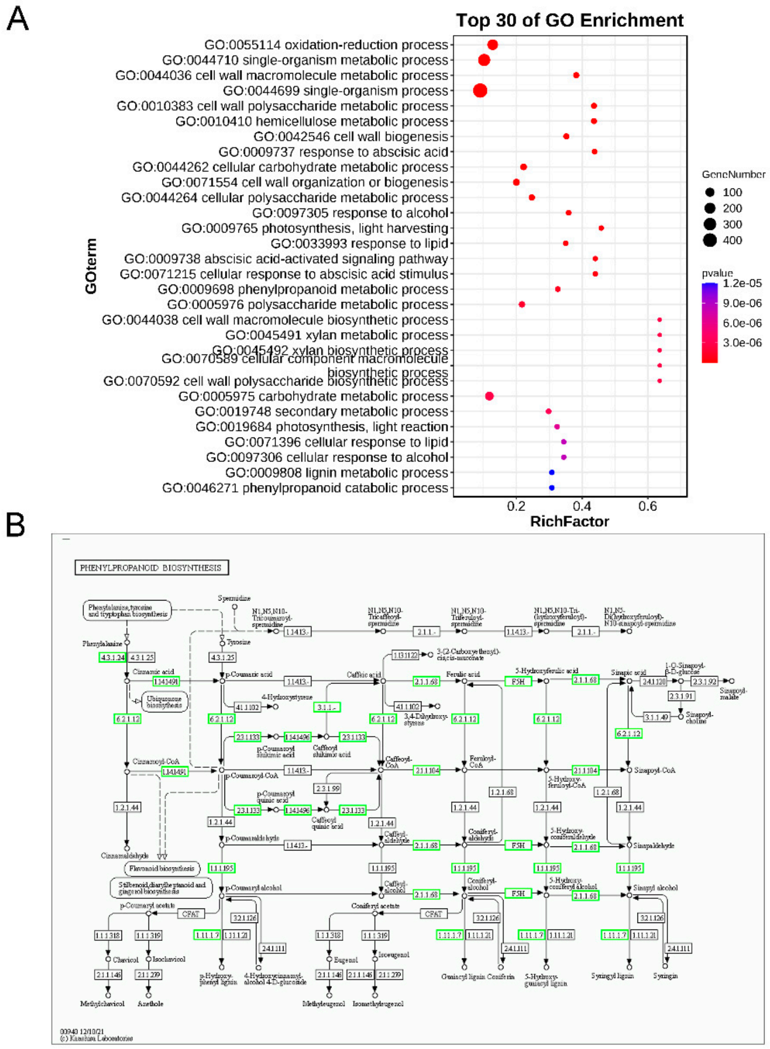

Supplemental Figure S4. GO enrichment analysis and KEGG enrichment analysis of genes co-induced by overexpression of FvVND4c and FvMYB46.

(A) GO enrichment analysis based on the overlapping differential expressed genes in response to overexpression of FvVND4c and FvMYB46. ($p < 0.05$, |log2(Fold change) >1.0 |).

(B) KEGG enrichment analysis based on the overlapping 348 genes co-induced by overexpression of FvVND4c and FvMYB46 showed that genes involved in phenylpropanoid biosynthesis pathways contribute to lignin and flavonoid synthesis were enriched.
